# Chelsea Hospital for Women

**Published:** 1915-11-13

**Authors:** Ilchester Helen

**Affiliations:** President of the Chelsea Hospital for Women.; President of the Ladies' Committee.


*To the Editor of* THE HOSPITAL.

### Chelsea Hospital for Women.

*To the Editor of* THE HOSPITAL.

SIR,—As the President of the Chelsea Hospital for Women and the President of its Ladies' Committee respectively, we beg that you will bring to the notice of your readers our urgent need of funds for the completion of the new hospital and the erection of its nurses' home. The contract for the new buildings was entered into before the war broke out, so that we have all the advantage of pre-war prices, but a sum of £35,000 is still required.

The usefulness of the institution will be greatly extended by the additional beds which will be provided. The new hospital is approaching completion, and when it is finished it will be possible to admit many poor sufferers who otherwise would have to be kept waiting a long time, or who would be altogether excluded. The facilities given at the hospital for the admission of the wives and near relations of our soldiers and sailors are, as we have occasion to know, proving a great boon both to them and to their relatives.

We regret to have to make this appeal at a time when so many claims are being made in connection with the war, but the matter is urgent. The work cannot be stopped; we have come to the end of our available resources, and the builders must be paid.

We shall be very grateful for any contributions (sent to Holland House, Kensington, W.) to add to the list of those who are giving us their generous support. Cheques should be crossed "London County and Westminster Bank, Sussex Place, S.W."

LONDONDERRY,
President of the Chelsea Hospital for Women.
HELEN ILCHESTER,
President of the Ladies' Committee.

